# Dental Students’ Perceptions of a Self-Directed Simulation-Based Learning Methodology (MAES©): A Pilot Study

**DOI:** 10.3390/dj14050305

**Published:** 2026-05-15

**Authors:** Sonia Guzmán, Alfonso García, María Ángeles Velló-Ribes, Olga Cortés

**Affiliations:** 1 Paediatric Dentistry, Department of Stomatology, Faculty of Medicine and Dentistry, University of Murcia, 30007 Murcia, Spain; ocortes@um.es; 2 Anesthesia and Resucitation, Faculty of Nursing, Catholic University of Murcia, Guadalupe, 30107 Murcia, Spain; agarcia@ucam.edu; 3 Paediatric Dentistry, Department of Stomatology, Faculty of Medicine and Dentistry, University of Valencia, 46010 Valencia, Spain; m.angeles.vello@uv.es

**Keywords:** dental education, simulation-based learning, self-directed learning, student satisfaction, students’ perceptions, pilot study, questionnaires, MAES© methodology

## Abstract

**Highlights:**

**What are the main findings?**

- The MAES© methodology resulted in high levels of student satisfaction, motivation, and perceived learning in dental simulation.
- Students highlighted active participation, teamwork, and structured debriefing as key elements of the learning experience.

**What are the implications of the main findings?**

- MAES© was perceived as a feasible and student-centered methodology for simulation-based learning in undergraduate dental education.
- The methodology may help support active learning, collaboration, and reflective practice in clinical training settings.

**Abstract:**

**Background/Objectives**: Simulation-based education is increasingly used in health sciences to promote active learning and the development of clinical and non-technical skills. However, its implementation in undergraduate dental education remains limited. This study aimed to explore dental students’ perceptions of the Self-Learning Methodology in Simulated Environments (MAES©) applied to high-fidelity simulation. **Methods**: A mixed-methods, cross-sectional pilot study was conducted with 80 fourth-year dental students enrolled in a Pediatric Dentistry course at a Spanish university. Quantitative data were collected using a validated satisfaction questionnaire (Cronbach’s alpha = 0.905), and descriptive statistics were performed. Qualitative data were obtained through open-ended questions and analyzed using inductive content analysis. **Results**: Students reported high levels of satisfaction, motivation, and perceived learning, with mean scores above 8.5 out of 10 across all evaluated dimensions. The facilitator’s role received the highest ratings. Qualitative analysis identified four main themes: perceived advantages of the methodology, increased engagement and participation, the value of structured debriefing, and areas for improvement related to group dynamics and performance-related stress. **Conclusions**: The MAES© methodology was well received and perceived as a feasible approach in dental simulation-based education. It may support student-centered learning, collaboration, and reflective practice, providing practical guidance for educators interested in implementing active learning strategies. As an exploratory pilot study conducted in a single institution, these findings should be interpreted cautiously and warrant further research.

## 1. Introduction

Simulation-based education has become an essential teaching strategy in health sciences, supporting the development of clinical competence, communication skills, and professional confidence [1,2,3,4]. However, its implementation in undergraduate dental education remains limited, particularly in some European contexts. Traditional dental education has historically relied on lectures, seminars, and laboratory-based training, with less emphasis on simulation-based methodologies. This approach may lead to gaps in active learning and patient safety competencies, which are key priorities in contemporary higher education [5].

Modern dental practice requires not only technical expertise but also the ability to manage complex clinical situations, communicate effectively with patients and families, and work collaboratively within healthcare teams [6]. Unlike many other health disciplines, dental education demands the simultaneous integration of psychomotor skills, behavioral management, communication, and rapid clinical decision-making in real-time patient care. These competencies are difficult to develop exclusively through traditional teaching approaches, highlighting the need for more active, student-centered, and experiential learning strategies in dental curricula.

Recent studies in dental education have highlighted the growing role of simulation-based learning in improving clinical reasoning, patient communication, and procedural confidence in undergraduate students [6,7]. High-fidelity simulation has been increasingly applied in pediatric dentistry and other dental specialties, showing positive effects on confidence, preparedness, and active engagement [6,7,8]. However, most available simulation models remain instructor-centered, offering fewer opportunities for self-directed learning, collaborative case design, and structured reflective debriefing, which are central components of the MAES© methodology.

The Self-Learning Methodology in Simulated Environments (MAES©) represents an innovative educational approach that integrates simulation, self-directed learning, and collaborative work [9]. Unlike traditional simulation models, MAES© places students at the center of the learning process by allowing them to select clinical scenarios, define learning objectives, and actively participate in case design and reflection, under the guidance of a facilitator. In particular, MAES© addresses the limited student autonomy and passive participation often observed in conventional simulation models by promoting active case construction, peer collaboration, and structured reflective debriefing. This approach has been associated with increased motivation, engagement, and perceived learning in other health disciplines, particularly in nursing and interprofessional education [9,10,11].

Despite these promising results, evidence regarding the application of MAES© in dental education remains scarce. Understanding how students perceive this methodology is essential for evaluating its feasibility and potential integration into dental curricula.

Therefore, the aim of this study was to explore dental students’ perceptions of the MAES© methodology implemented through high-fidelity simulation. Specifically, the study sought to assess students’ satisfaction, motivation, and perceived learning, as well as their experiences with teamwork and facilitator support. It was hypothesized that students would report positive perceptions related to self-directed learning, collaboration, and the integration of clinical competencies.

## 2. Materials and Methods

### 2.1. Study Design and Ethical Considerations

A convergent mixed-methods, cross-sectional, descriptive pilot study was conducted to explore dental students’ perceptions of the MAES© simulation-based learning methodology. Quantitative and qualitative data were collected concurrently, analyzed independently, and integrated during interpretation to provide complementary insights into students’ perceptions.

The study was approved by the Institutional Review Board of the University of Murcia (protocol code M10/2023/087; approval date: 19 December 2023). All participants provided informed consent prior to participation. The study was conducted in accordance with the Declaration of Helsinki, and all data were anonymized to ensure confidentiality.

### 2.2. Setting and Educational Intervention

**Figure 1:**
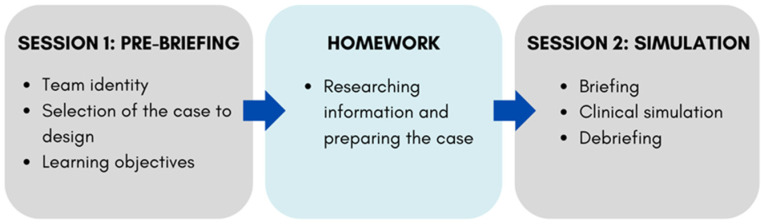
Summary diagram of the sessions organized for simulation using MAES©.

The study was carried out within the Pediatric Dentistry course at the University of Murcia. MAES© is structured with at least two in-person sessions and one week dedicated to homework for case preparation (Figure 1).

During the initial session, a conducive and psychologically safe environment was established for students and facilitators [9], facilitating the creation of autonomous work teams with a distinct group identity. The facilitators introduced a variety of topics in an engaging way, using videos, news clips, and other media, all of which were related to key competencies essential for the pediatric dentistry course. Each team was free to select a topic to develop a simulation scenario. After assigning the topics, a collective brainstorming session was held to define the learning objectives and identify the technical and non-technical skills each group wished to acquire in connection to their scenario. Following the initial session, the groups were given at least one week to develop their cases based on the guidelines outlined during the pre-briefing. While the teams worked autonomously, the professor remained available to address any questions or concerns. Before the simulations were executed, the professor reviewed all cases to ensure the absence of clinical inconsistencies and to confirm adherence to the required scientific standards and difficulty level. Each team designed a scenario, but rather than performing their own case, they participated as the primary actors in scenarios developed by other teams. Table 1 provides an overview of the scenarios and the competencies selected by the students.

In the second session, the simulation exercise was carried out. The teams responsible for designing the cases began with a brief overview, providing only essential background information and context to the entire class. Students from a different team were selected to experience the simulated clinical scenario without additional details.

Those who had created the case took on the roles of standardized patients, assuming roles such as parent, child, or caregiver, while the students assigned to the case acted as the dentist and assistant. The professor overseeing the session did not intervene during the simulation but monitored its technical and logistical progression, noting the behaviors and competencies displayed by the students conducting the simulation.

The remaining students observed the simulation from an adjacent debriefing room via a television feed. They recorded relevant observations in their notepads and played an active role in the subsequent debriefing session [9,10]. During the structured debriefing, which followed the phases of reaction, analysis, and summary, the students who had designed the case presented the scientific evidence they had identified as relevant competencies during the pre-briefing session.

Finally, the entire group engaged in a collective summary, discussing what had been learned and reflecting on the key takeaways from the simulation.

### 2.3. Participants

The target population consisted of two consecutive cohorts of fourth-year dental students enrolled in the Pediatric Dentistry course during the 2023–2024 and 2024–2025 academic years (*n* = 80).

Inclusion criteria were: (1) enrollment as a fourth-year dental student and (2) prior consent to participate in research activities. Exclusion criteria included absence during data collection, withdrawal from the course, or participation in exchange programs.

All students had prior exposure to low-fidelity simulations but had not previously participated in high-fidelity simulation activities.

### 2.4. Variables and Data Collection

The primary outcome variables included five dimensions: advantages, motivation, student satisfaction, facilitator effectiveness, and teamwork. These dimensions were assessed using 20 items on a 10-point Likert scale ranging from 1 (totally disagree) to 10 (absolutely agree).

Quantitative data were collected through an online questionnaire administered via Google Forms after the simulation sessions. The questionnaire consisted of 20 items adapted from a previously published MAES© evaluation instrument used in simulation-based learning research [10]. Internal consistency of the questionnaire was assessed in the current study sample, obtaining a Cronbach’s alpha coefficient of 0.905 for the full scale.

Qualitative data were obtained through open-ended questions included in the survey, allowing students to provide feedback on strengths, weaknesses, and suggestions for improvement.

### 2.5. Study Size

The study included a total of 80 dental students, representing 100% of the cohort exposed to the MAES© methodology. As a pilot study, the sample size was considered appropriate to explore feasibility, acceptability, and preliminary outcomes [12,13].

The inclusion of a complete cohort minimized selection bias and reduced variability associated with group heterogeneity. No a priori sample size calculation was performed due to the exploratory nature of the study.

### 2.6. Statistical Analysis

Quantitative data were analyzed using the Statistical Package for the Social Sciences (SPSS^®^) version 21. Descriptive statistics, including means, standard deviations, frequencies, and percentages, were calculated. The reliability of the questionnaire was assessed using Cronbach’s alpha coefficient.

Qualitative data were analyzed using an inductive content analysis approach [13]. Two researchers independently reviewed and coded the qualitative responses. Codes were grouped into themes through iterative discussion until consensus was reached.

Inferential statistics were not performed due to the exploratory and descriptive nature of this pilot study.

### 2.7. Data Availability

The data presented in this study are available on request from the corresponding author. Data are not publicly available due to privacy and ethical restrictions.

## 3. Results

### 3.1. Participant Characteristics

A total of 80 fourth-year dental students participated in the study. The mean age of the participants was 22.5 years (range: 21–25 years). Of the participants, 60% (*n* = 48) were female and 40% (*n* = 32) were male. All participants belonged to the same academic level and had completed at least three years of undergraduate dental education prior to participating in the simulation-based learning activities.

### 3.2. Quantitative Results

Descriptive statistics were calculated to analyze students’ perceptions across the evaluated dimensions: advantages, motivation, satisfaction, facilitator effectiveness, and teamwork.

**Figure 2:**
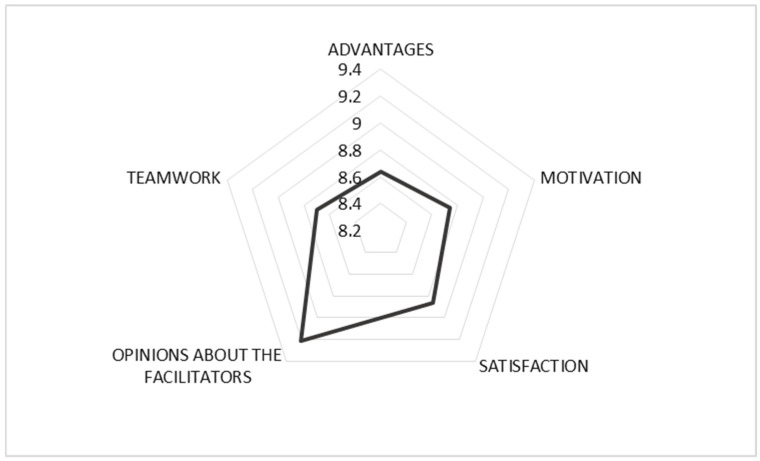
Illustrates the average scores across the evaluated dimensions.

As shown in Table 2, all dimensions received high scores, with mean values exceeding 8.5 out of 10. The highest ratings were observed in the facilitator effectiveness dimension, indicating the importance of guidance and support during the simulation process (Figure 2). The complete questionnaire structure, including dimensions, item scores, and internal consistency values, is available in the Supplementary Materials (Table S1).

The quantitative findings showed consistently high scores across all evaluated dimensions. The most highly rated aspects were facilitator effectiveness, student satisfaction, and motivation, highlighting the perceived value of guided support, active participation, and collaborative learning within the MAES© methodology.

### 3.3. Qualitative Results

Qualitative content analysis of the open-ended responses identified four main themes.

#### 3.3.1. Perceived Advantages of the MAES© Methodology

Students highlighted that the methodology improved their understanding of clinical roles and situations. They emphasized the opportunity to engage in realistic scenarios and develop clinical reasoning skills: “… it helps better to understand the roles of dentist/family/patient…”, “I consider it necessary to have a first contact with what real life is like to help us face various future situations and not feel overwhelmed,” “… we put ourselves in the role of complex cases…”, “it provides a different approach than usual and undoubtedly helps to break the monotony and seriousness of classical training.” Additionally, students reported increased involvement in their own learning process due to active participation in case design: “you get involved by searching for information yourself, and you learn more.”

#### 3.3.2. Engagement and Active Learning

Participants described the methodology as dynamic and engaging, noting that it maintained their attention and made learning more enjoyable. Many students emphasized that this approach differed from traditional lectures by promoting active participation: “It’s more enjoyable than normal class, and we get straight to the point of information.” “… an entertaining way to learn…”, “good experience, fun and useful; the proof is that I haven’t checked my phone once.”

#### 3.3.3. Value of Debriefing

The debriefing sessions were considered a key component of the learning process. Students reported that group discussions facilitated reflection, improved understanding, and encouraged participation. The opportunity to share perspectives was perceived as beneficial for consolidating knowledge: “I like that it is considered that there are other ways of learning”, “by everyone participating, it’s easier to lose the fear of speaking in public”, “the debriefing part has been the most important because the contributions of the team and even those of the peers are generally quite constructive and focus on solving or improving very specific and clear aspects.”

#### 3.3.4. Areas for Improvement

Some students identified challenges related to group dynamics, including unequal participation among team members. Others reported feelings of stress during high-fidelity simulation scenarios: “… sometimes it’s a bit uncomfortable because you feel pressure knowing that the teachers and peers are there”. Suggestions for improvement included better timing of sessions and combining MAES© with other methodologies focused on technical skills: “it would be interesting to conduct this practice after some time, to observe if the acquired knowledge from the first simulation could be absorbed…” or “I think it would be very interesting for the teaching team to also participate with some kind of role to guide further certain situations that usually arise in day-to-day practice…”

## 4. Discussion

The findings of this study suggest that the MAES© methodology was positively perceived by dental students as a student-centered and engaging learning strategy. Participants valued the opportunity to actively construct clinical cases, collaborate with peers, and participate in structured reflective debriefing. These findings reinforce the potential of MAES© to promote active learning, teamwork, and reflective practice in undergraduate dental education, which are essential competencies for clinical training.

The results of this study suggest that the MAES© methodology can significantly contribute to improving students’ learning experiences in dental education by promoting active, self-directed learning. These quantitative findings were reinforced by the qualitative results, in which students highlighted the realism of the simulated scenarios, the active role they assumed during case preparation, and the educational value of structured debriefing. These themes help explain the high levels of satisfaction and motivation observed and support the perceived relevance of MAES© as an active learning methodology. The high ratings for realism and facilitator effectiveness underscore the importance of creating psychologically realistic simulations and providing strong guidance during these sessions [14,15]. Despite its success, some limitations, such as group dynamics where students felt unequal participation, indicate the need for further refinement. Given the small sample size and localized context, these findings should be interpreted cautiously, recognizing that they provide promising preliminary insights but warrant further investigation in more extensive and diverse settings.

The positive outcomes observed in this study are consistent with findings from other fields such as nursing and interprofessional education, where MAES© has been shown to increase motivation, satisfaction, and collaborative learning [9,11,16,17]. Similar findings have also been reported in dental education, where simulation-based methodologies have been associated with improved student confidence, engagement, and clinical preparedness, particularly in pediatric and preclinical training setting [6,7,8]. In particular, the high ratings for facilitator effectiveness align with previous [18,19,20,21], reinforcing the critical role of instructors in simulation-based education. Moreover, the use of group dynamics and peer learning, which are key features of MAES©, parallels findings in problem-based learning methodologies, further supporting its potential to foster autonomy and motivation. However, this is the first study to evaluate the use of MAES© in dental education, making direct comparisons to other dental studies difficult.

This study has several limitations. First, as a pilot study conducted in a single institution, the sample size limits the generalizability of the findings. Second, the study relied on self-reported measures of perception and satisfaction, which may introduce self-report bias and social desirability bias, as students may have felt inclined to evaluate the methodology positively. In addition, the novelty of the MAES© methodology may have contributed to participant enthusiasm bias, potentially influencing the high satisfaction scores observed. Furthermore, the absence of objective performance indicators and longitudinal follow-up limits the ability to assess the long-term educational impact of the intervention. Future studies should incorporate comparative designs, longitudinal assessments, and objective clinical performance outcomes to strengthen the evidence regarding the educational value of the MAES© methodology.

## 5. Conclusions

The findings of this pilot study suggest that dental students perceived the MAES© methodology positively, particularly regarding active learning, teamwork, and reflective practice. The methodology was considered feasible and well accepted within undergraduate dental education and may represent a valuable student-centered approach for simulation-based teaching. However, given the exploratory and perception-based nature of this study, further research incorporating objective learning outcomes, comparative designs, and longitudinal assessments is needed to better understand its educational impact.

## Figures and Tables

**Table 1:** The corresponding MAES© scenarios and learning objectives for each chosen team identity.

Team Identity	Case to Design	Learning Objectives
“PEAKY BLASTOS”	Caries and long-term breastfeeding	How long should breastfeeding be maintained? Repercussions of prolonged breastfeeding Benefits of prolonged breastfeeding Behaviour management (psychological profile)
“THE CROWN”	Ectopic eruption	Etiology and diagnosis Treatment: when should we take action? Approach to an anxious and distrustful mother
“THE GUTAPERCHOS”	Oral thrush	Transmission of thrush Treatment of the disease Consequences of the infection Importance in nutrition
“THE APOLONIOS”	Down Syndrome	Behavior management Medication and pathology Prevention of sequelae (oral hygiene, diet control…)

**Table 2:** Descriptive statistics of the dimensions of the questionnaire and Cronbach’s alpha for each dimension.

*Dimensions, N = 80*	*Mean*	*SD*	*α*
*Advantages*	8.64	1.31	0.902
*Motivation*	8.74	1.45	0.896
*Satisfaction*	8.86	1.13	0.899
*Opinions about the facilitators*	9.21	0.707	0.901
*Teamwork*	8.70	1.41	0.905

## Data Availability

The data presented in this study are available on request from the corresponding author. The data are not publicly available due to privacy and ethical restrictions.

## Supplementary Materials

The following supporting information can be downloaded at: https://www.mdpi.com/article/10.3390/dj14050305/s1, Table S1: Descriptive statistics and internal consistency of questionnaire dimensions and items.

## Abbreviations

The following abbreviations are used in this manuscript:

MAES©	Self-Learning Methodology in Simulated Environments
