# Supplementary material for: Dental Students’ Perceptions of a Self-Directed Simulation-Based Learning Methodology (MAES©): A Pilot Study
Source: Dent J (Basel). 2026 May 15;14(5):305. doi: 10.3390/dj14050305 (PMC13206454; doi:10.3390/dj14050305)
Supplement: Supplementary file 1 [file dentistry-14-00305-s001.zip › dentistry-4261856-supplementary.pdf]

Table S1.

DIMENSIONS and ITEMS, N=80	***Mean***	***SD***	***α***
Advantages	**8,64**	**1,31**	**0,902**
"The MAES© methodology enhances my learning capacity."	9,06	1,027	0,902
"Learning with MAES© stimulates my creativity and ability to research and solve problems."	8,91	0,707	0,896
"I feel more confident with MAES© than other methodologies when I practice with cases in the simulation room."	7,89	0,820	0,905
"I think I acquired more knowledge with MAES© than other high-fidelity simulation methodologies."	8,43	0,707	0,903
"The debriefing is more prosperous, and I prepare it better when working with the MAES© method."	8,46	0,707	0,906
"Although it takes longer to prepare the case, I go deeper into the knowledge of the clinical situation."	8,71	0,707	0,907
"MAES© makes me feel more prepared to face a real situation, although I have previously worked in simulation."	8,89	2,83	0,904
"My degree of responsibility in my learning is high, so I learn more."	8,80	0,707	0,901
"I think my knowledge is more efficient with MAES© than other simulation methodologies."	8,57	1,41	0,894
**Motivation**	**8,74**	**1,45**	**0,896**
"The freedom to choose cases stimulates my learning, and my role is more active."	8,69	4,24	0,894
"I feel more motivated with MAES© than other clinical simulation methodologies."	8,63	1,41	0,895
"Promoting group identity in MAES© sessions (forming teams with a nickname and a group spirit, for example) increases the students' motivation."	8,91	0,707	0.900
**Satisfaction**	**8,86**	**1,13**	**0,899**
"I prefer to learn more often using the MAES© simulation methodology."	8,86	1,41	0,900
"The effort that the MAES© methodology requires from the students is worth it."	8,74	0,707	0,897
"Rate your level of satisfaction with the MAES© methodology."	9,00	1,41	0,900
Opinions about the facilitators	**9,21**	**0,707**	**0,901**
"The facilitators are sufficiently prepared to work with MAES© learning groups."	9,23	0,707	0,903
"The responsibility for learning lies with the students, and the facilitator plays a vital but secondary role."	9,09	0,707	0,902
"I consider that the role of the teacher is crucial in the initial motivation of the simulation groups that work with MAES© rather than in the transmission of knowledge."	9,31	0,707	0,898
Teamwork	**8,70**	**1,41**	**0,905**
"Thanks to the group work promoted by MAES©, the group acquires more knowledge than other simulation methods."	8,74	1,41	0,896
"The process of case selection, design, and preparation encourages teamwork."	8,66	1,41	0,914
